# Supplementary material for: Microbial community organization designates distinct pulmonary exacerbation types and predicts treatment outcome in cystic fibrosis
Source: Nat Commun. 2024 Jun 7;15:4889. doi: 10.1038/s41467-024-49150-y (PMC11161516; doi:10.1038/s41467-024-49150-y)
Supplement: Supplementary file 1 — Supplementary information [file 41467_2024_49150_MOESM1_ESM.pdf]

**Microbial community organization designates distinct pulmonary exacerbation types and predicts treatment outcome in cystic fibrosis.**

**Supplementary Information**

**Figure S1**

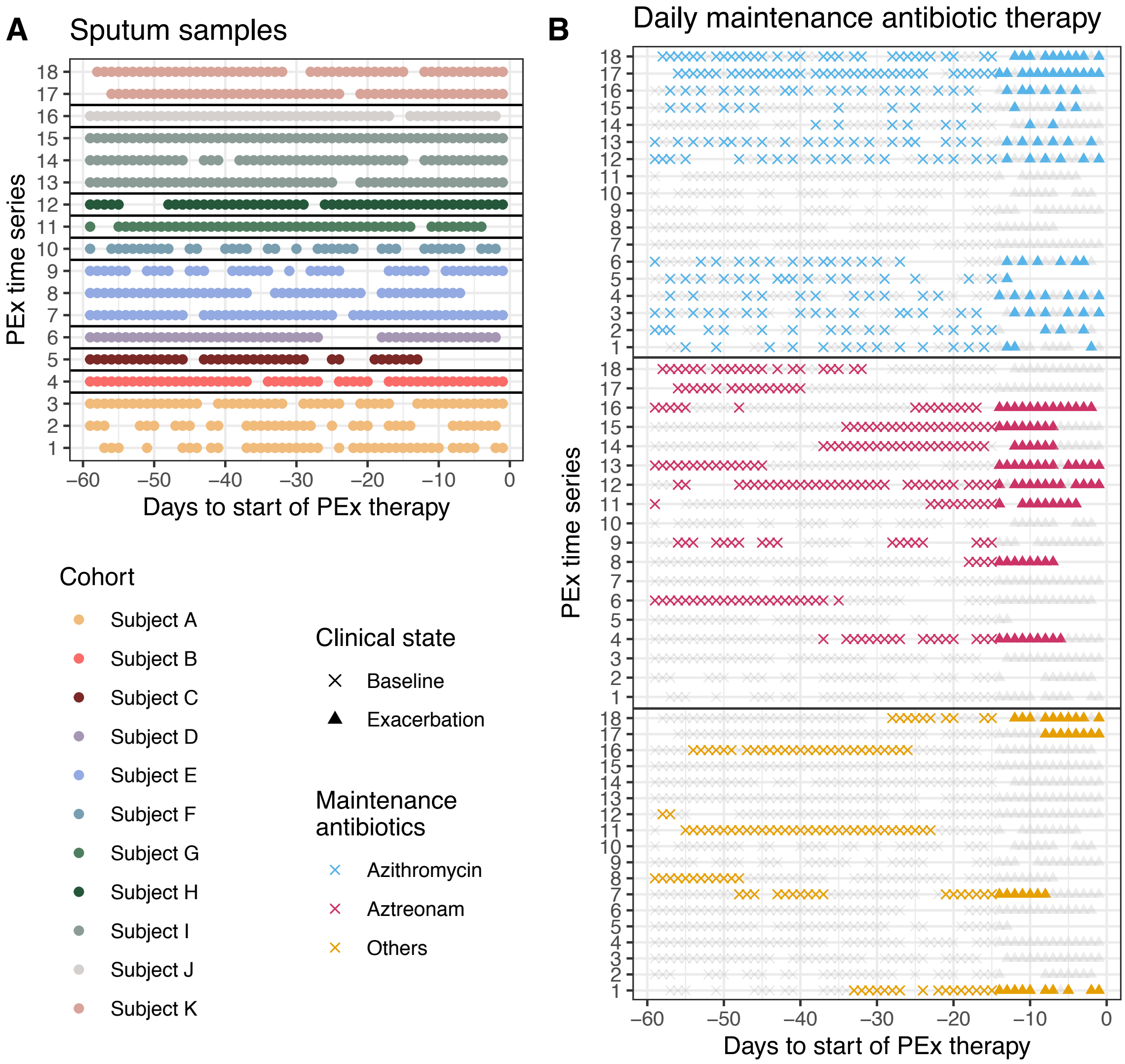


**Fig. S1 PEx time series and maintenance antibiotic therapy. a** Sputum samples collected during the 60 days prior to the start of antibiotic treatment for PEx are shown by subject for each of the 18 PEx time series. The numbers $n$ of samples for each of the 11 study subjects are 126, 53, 38, 50, 143, 37, 51, 51, 168, 56, and 107

for subjects A-K, respectively. **b** Maintenance antibiotic therapy per day during the PEx time series for each subject is shown. Samples collected after the start of antibiotic therapy for PEx were not included in the analyses.

## Figure S2

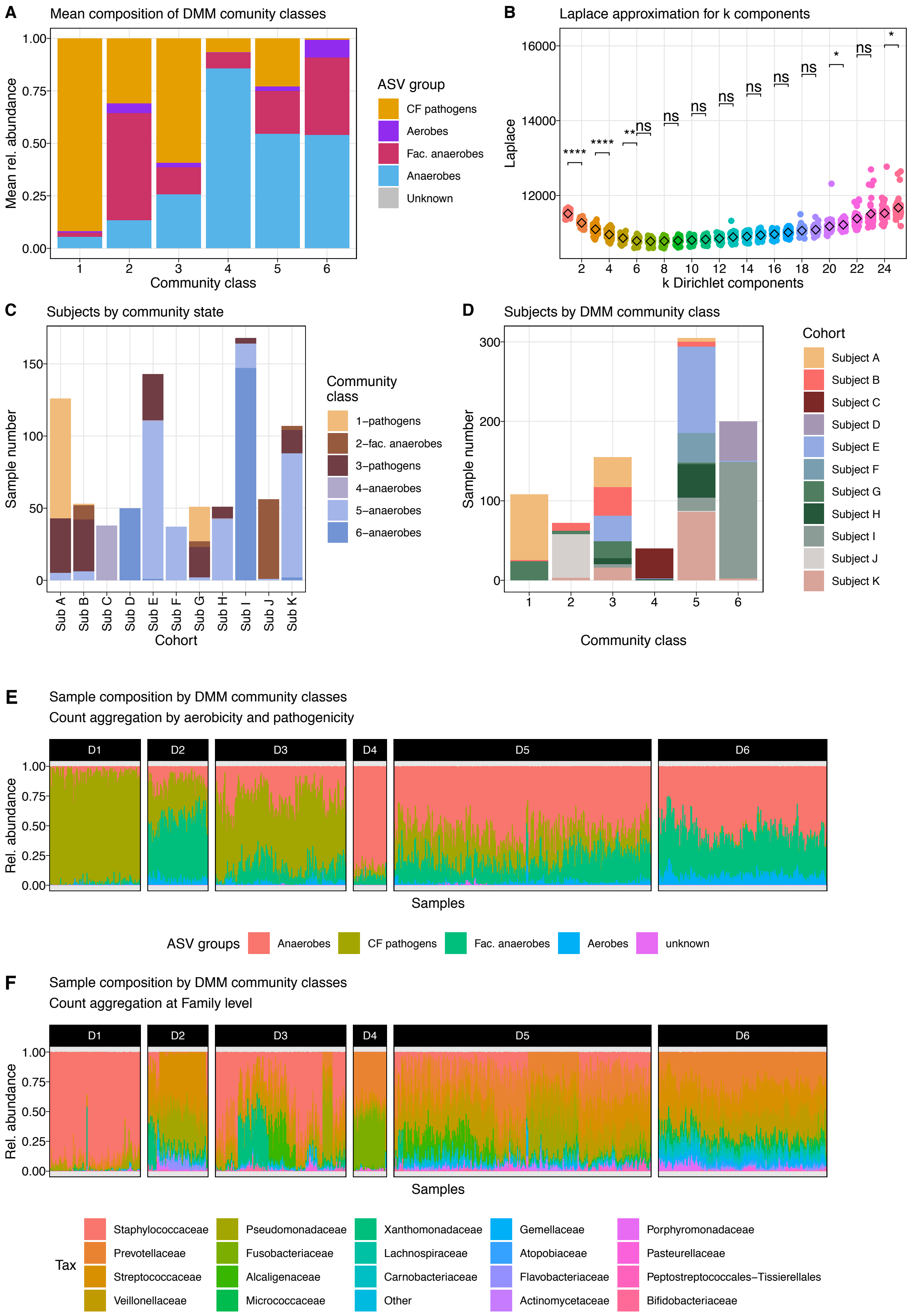
A
Mean composition of DMM comunity classes
Mean rel. abundance
Community class
ASV group
CF pathogens
Aerobes
Fac. anaerobes
Anaerobes
Unknown
B
Laplace approximation for k components
Laplace
k Dirichlet components
C
Subjects by community state
Sample number
Cohort
Community class
1–pathogens
2–fac. anaerobes
3–pathogens
4–anaerobes
5–anaerobes
6–anaerobes
Sub A
Sub B
Sub C
Sub D
Sub E
Sub F
Sub G
Sub H
Sub I
Sub J
Sub K
D
Subjects by DMM community class
Sample number
Community class
Cohort
Subject A
Subject B
Subject C
Subject D
Subject E
Subject F
Subject G
Subject H
Subject I
Subject J
Subject K
E
Sample composition by DMM community classes
Count aggregation by aerobicity and pathogenicity
D1
D2
D3
D4
D5
D6
Rel. abundance
Samples
ASV groups
Anaerobes
CF pathogens
Fac. anaerobes
Aerobes
unknown
F
Sample composition by DMM community classes
Count aggregation at Family level
Rel. abundance
Samples
Tax
Staphylococcaceae
Prevotellaceae
Streptococcaceae
Veillonellaceae
Pseudomonadaceae
Fusobacteriaceae
Alcaligenaceae
Micrococcaceae
Xanthomonadaceae
Lachnospiraceae
Carnobacteriaceae
Other
Gemellaceae
Atopobiaceae
Flavobacteriaceae
Actinomycetaceae
Porphyromonadaceae
Pasteurellaceae
Peptostreptococcales–Tissierellales
Bifidobacteriaceae

**Fig. S2 Identification of microbial community classes. a** Composition of six Dirichlet multinomial model (DMM) components ($n = 880$). DMM was inferred at the level of ASV groups: strictly aerobic, strictly anaerobic, facultatively anaerobic, conventional CF pathogens, and unknown oxygen requirement. **b** Identification of best DMM model. A total of $n = 36$ models, each with one to 25 components, was inferred and the Laplace approximations of each are presented together with stepwise comparison of means among models with k components (two-sided Wilcoxon, $\mathrm{p}_{1\ \mathrm{vs}\ 2} = 9.7\mathrm{e} - 16$, $\mathrm{p}_{3\ \mathrm{vs}\ 4} = 3.4\mathrm{e} - 5, \mathrm{p}_{5\ \mathrm{vs}\ 6} = 2.7\mathrm{e} - 3, \mathrm{p}_{6\ \mathrm{vs}\ 7} = 7.2\mathrm{e} - 1, \mathrm{p}_{20\ \mathrm{vs}\ 21} = 1.9\mathrm{e} - 2, \mathrm{p}_{24\ \mathrm{vs}\ 25} = 1.0e - 2$, without multiple testing correction). The first statistically non-significant change of Laplace was used as a cutoff criterion ($k = 6$). The diamond symbol depicts the mean value for each DMM component. **c** Distribution of DMM community states across the subject cohort ($n = 880$). Sample number by subject is depicted. **d** Distribution of subjects across DMM components ($n = 880$). Sample number by DMM is depicted. **e** Sample-wise community compositions aggregated by ASV groups and presented by DMM community classes (D1-6), as used for downstream analyses ($n = 880$). **f** Original sample-wise community compositions with ASVs aggregated at Family level (Tax) and grouped by DMM community classes (D1-6). Top 20 taxa are indicated ($n = 880$).

## Figure S3

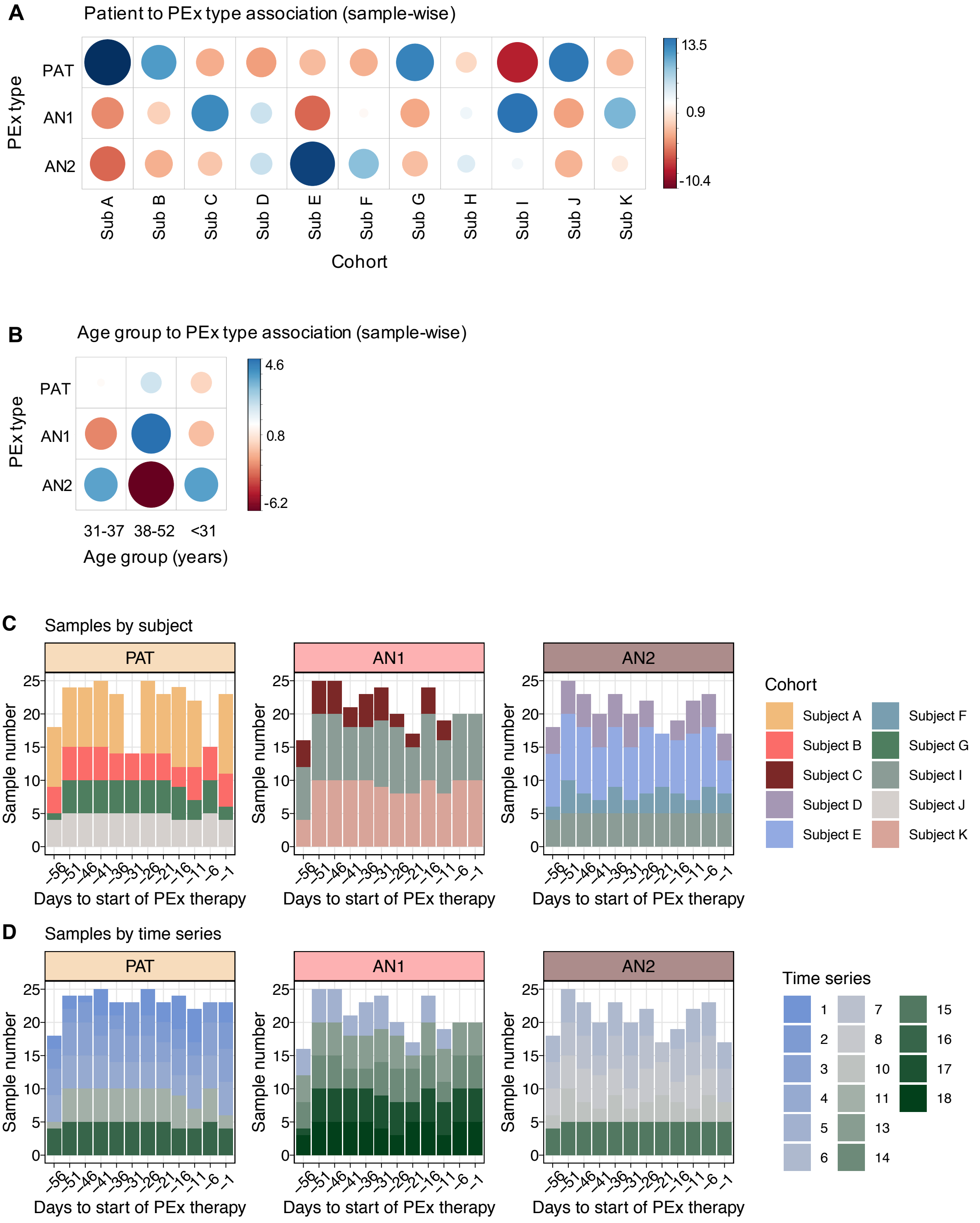


**Fig. S3 Sample distributions by PEx types, subjects, age groups and time series** ($n_{\text{PAT}} = 286, n_{\boldsymbol{AN1}} = 254, n_{\text{AN2}} = 249$). **a** Spearman rank association of PEx types and subjects ($S_A$-$S_K$). $\chi^2$testing performed on samples in respective

categories, standardized residuals shown ($\chi^2 = 700, \mathrm{p} < 2.2\mathrm{e} - 16$). **b** Spearman rank association of PEx types and age groups (<31, 31-37, 38-52 years). $\chi^2$testing performed on samples in respective categories, standardized residuals shown ($\chi^2 = 42.5, \mathrm{p} < 1.3\mathrm{e} - 8$). **c** Sample numbers as distributed across PEx types by subjects and time to PEx treatment as used in downstream analyses. Data aggregated by 5 days. **d** Sample numbers as distributed across PEx clusters by time series and time to PEx antibiotic treatment as used in downstream analyses. Data aggregated by 5 days.

## Figure S4

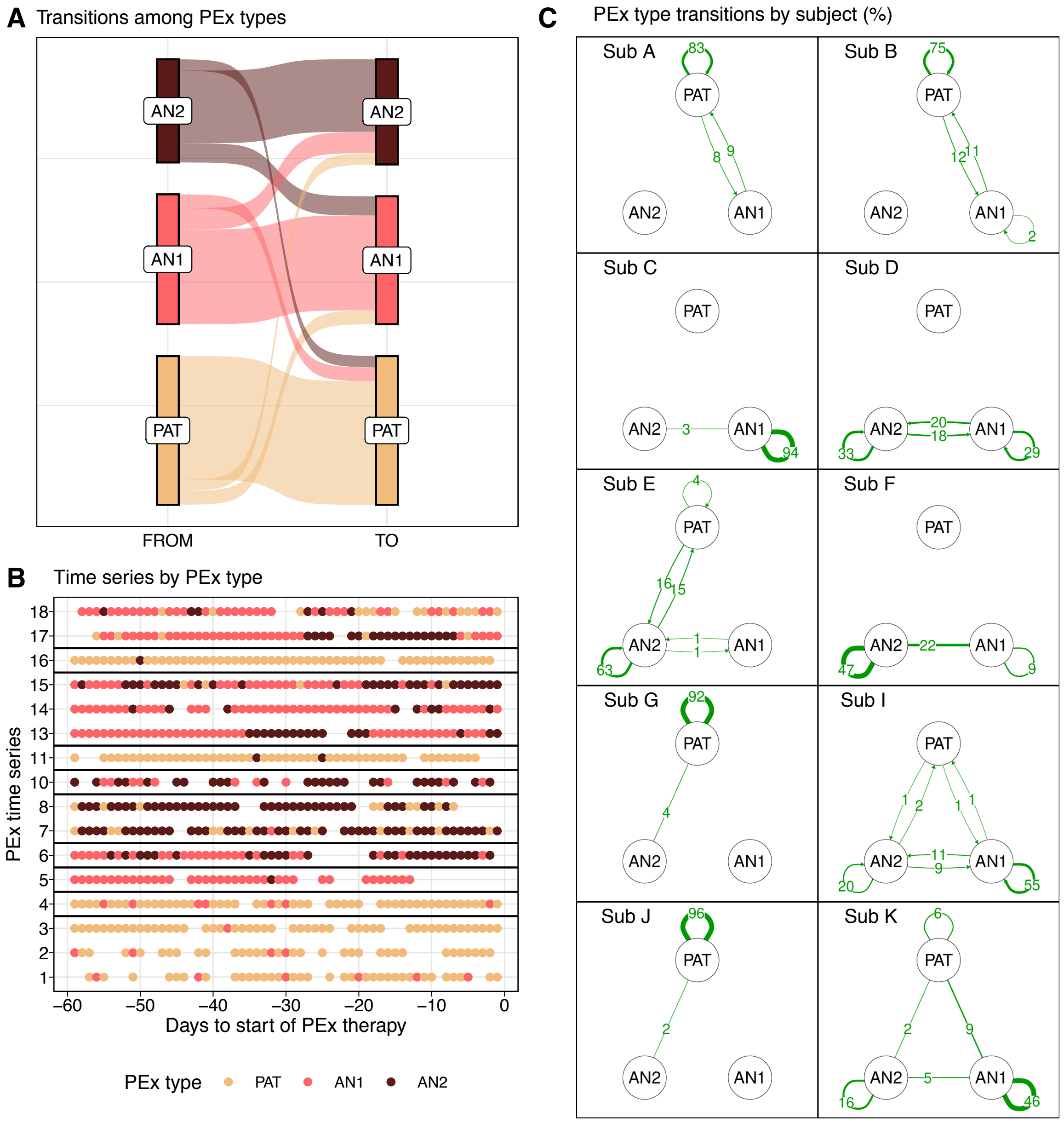


**Fig. S4 Transition profiles between PEx types.** Used samples distribute to PEx types as $n_{\mathrm{PAT}} = 309, n_{\mathrm{AN1}} = 266, n_{\mathrm{AN2}} = 214$. **a** Sankey plot depicting transition events between pairs of consecutive samples within individual PEx time series. A particular PEx type can be entered from the previous sample with different or same PEx type and can transition into the PEx type of the subsequent sample. Transition events distribute for AN2: 11% to PAT, 70% within AN2, 19% to AN3; for AN1: 11% to PAT, 16% to AN2, 73% within AN1; for PAT 83% within PAT, 8% to AN2, 9% to AN1. **b** Samples with respective PEx type classification shown as individual time

series. **c** Transition graphs reporting proportion (%) of PEx type maintenance (self-loops) or switch (edges) by individual subject (Sub A – Sub K). Edges labelled and sized according to transition percentage, undirected edges indicate the same amount of transitions in both directions.

## Figure S5

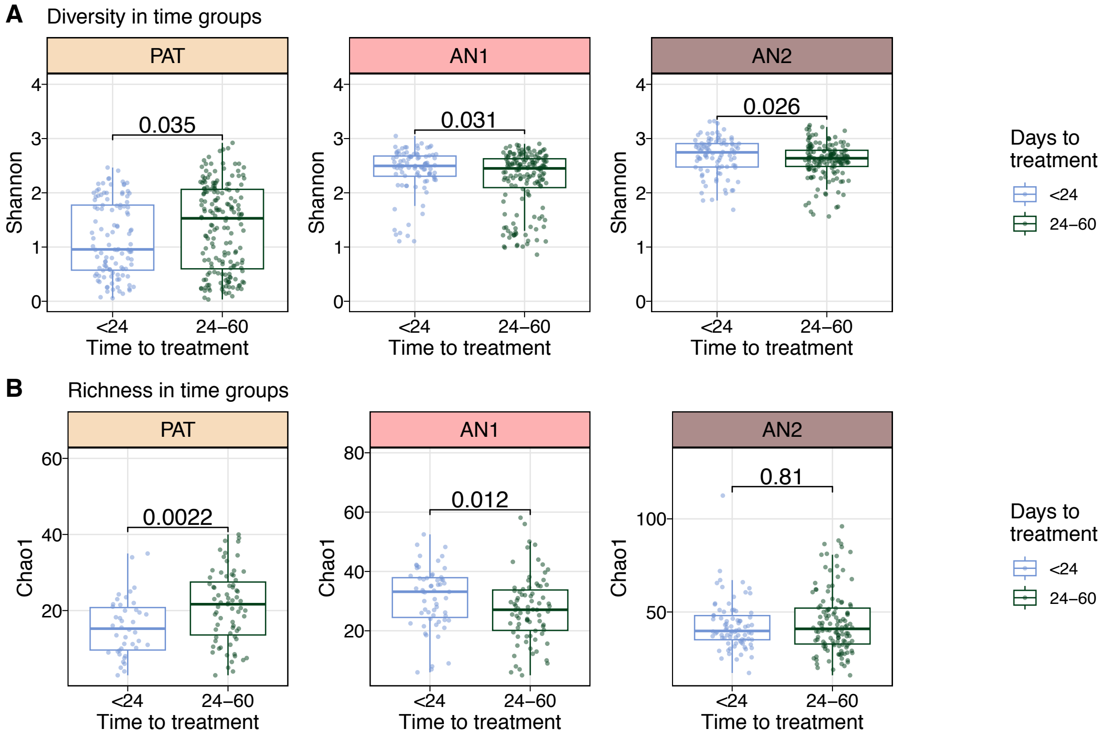


**Fig. S5 Time evolution of microbiome diversity and richness by time group and PEx type** (PAT, AN1, AN2, $n_{\text{PAT}} = 286, n_{\boldsymbol{AN1}} = 254, n_{\text{AN2}} = 249$). **a** Shannon diversity in microbiomes collected either <24 days or 24-60 days before PEx antibiotic treatment. Exact p values of two-sided Wilcoxon tests depicted. **b** Chao1 richness in microbiomes collected either <24 days or 24-60 days before PEx antibiotic treatment. Exact p values of two-sided Wilcoxon tests depicted.

Boxes in **b** and **c** depict the interquartile range; the horizontal line represents the median value. Whiskers extend to the minimum and maximum values, excluding outliers, defined as 1.5 times the interquartile range.

## Figure S6

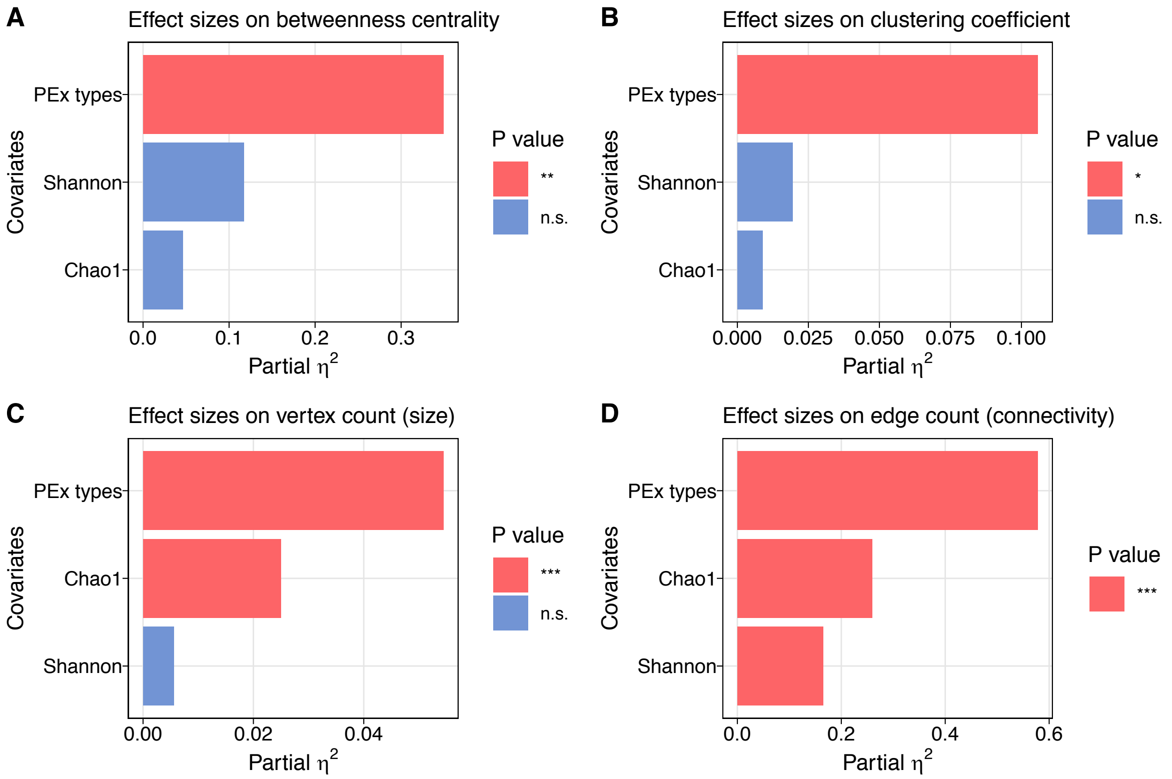


**Fig. S6 Effect sizes of PEx types, and covariates Shannon diversity and Chao1 richness on co-occurrence topology** ($n = 589$). **a-d** Independent mixed effect models (LMM) were implemented, statistical significance of covariates determined (ANOVA) and the partial effect sizes $\eta^2$ of covariates on betweenness centralities **(a)**, clustering coefficients **(b)**, vertex counts **(c)** and edge counts **(d)**, assessed and visualized. The analyses were performed on the largest components of co-occurrence networks. Levels of statistical significance displayed by color code in legend (significance codes: $p < 0.001$(***); $p < 0.01$(**); $p < 0.05$(*); n.s. not significant). Exact p values (ANOVA, type III) for **a** $p_{PEx} = 3.4e-3$, $p_{Shannon} = 1.3e-1$, $p_{Chao1} = 2.4e-1$, for **b** $p_{PEx} = 4.8e-2$, $p_{Shannon} = 2.5e-1$, $p_{Chao1} = 2.1e-1$, for **c** $p_{PEx} = 2.8e-7$, $p_{Shannon} = 7.5e-2$, $p_{Chao1} = 1.2e-4$, and for **d** $p_{PEx} = 7.2e-11$, $p_{Shannon} = 1.2e-4$, $p_{Chao1} < 2.2e-16$.

**Figure S7**

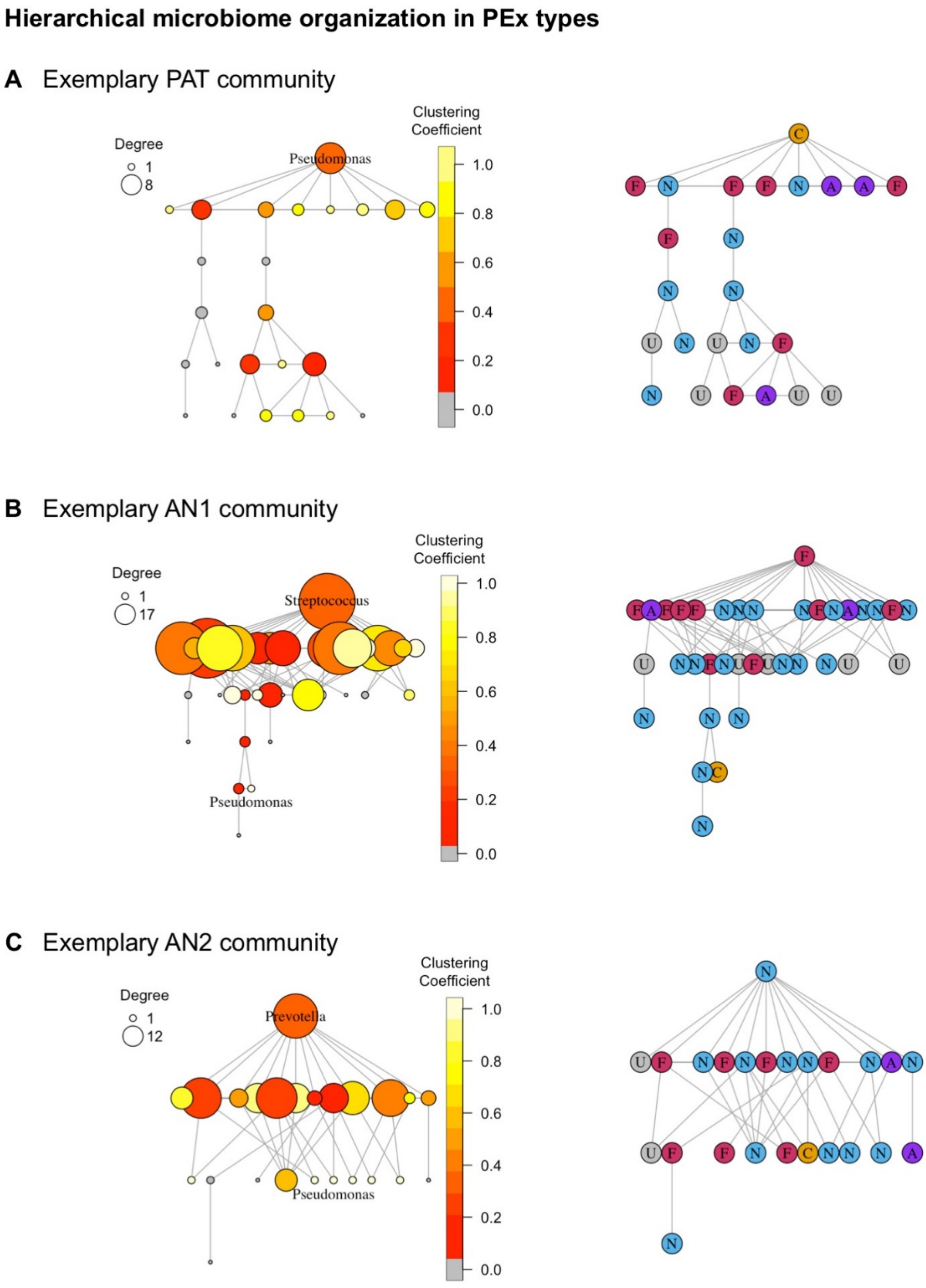


**Fig. S7 Hierarchical organization of microbiomes in PEx types reveals different positioning of pathogens in three networks representative for PEx types. a-c** Co-occurrence network layouts of exemplary PEx type communities

were designed with Sugiyama algorithm for hierarchical networks[1]. **left** Node size was scaled according to its degree $k$, node color according to local clustering coefficient $\mathrm{CC}$. The most hierarchical position of node $i$ in the community was identified by highest degree $k_i$ and lowest clustering coefficient ($\mathrm{CC_i} > 0$). Present pathogens (*Pseudomonas*), as well as ASVs locating to hierarchical positions with highest frequency are indicated by PEx type. **right** Corresponding graphs with node labels according to ASV groups. Pathogens were depicted as C/orange, aerobes as A/purple, facultative anaerobes as F/pink, anaerobes as N/soft blue, ASVs with unknown oxygen requirements as U/light grey.

# Tables

## Table S1 Inclusion criteria and analyses for samples and PEx time series.

| Inclusion criteria | Samples | Subjects | Time series | Samples by subject mean (sd) | Samples by time series mean (sd) | Time series by subject mean (sd) | Analyses |
|---|---|---|---|---|---|---|---|
| ≥ 60 days w/o acute treatment | 880 | 11 | 18 | 80.0 (46.9) | 48.9 (7.3) | 1.6 (0.9) | DMM<br>PCA<br>clustering<br>NW inference<br>noise colors |
| ≥ 60 days w/o acute treatment,<br>≥ 40% time series coverage by PEx cluster | 789 | 10 | 16 | 78.9 (44.4) | 49.3 (7.4) | 1.6 (0.8) | time series classification<br>temporal evolution<br>community turnover |

## Table S2 Inclusion criteria and analyses for networks (NW).

| Inclusion criteria | NWs | Subjects | NWs by PEx cluster PAT; AN1; AN2 | NWs by subject mean (sd) | Analyses |
|---|---|---|---|---|---|
| samples from classified time series, NW classification by majority vote of sample cluster association | 589 | 10 | 222; 192; 175 | 58.9 (31.3) | NW classification<br>topological analyses<br>taxonomic hierarchy<br>treatment simulation |

## Table S3 Kolmogorov Smirnov test to contrast cumulative distributions of network properties before and after simulated pathogen removal.

| Kolmogorov Smirnov distribution test | | | |
|---|---|---|---|
| Network property | Test cases | D statistic | p value |
| no. of nodes | PAT vs AN1 | 0.33 | 2.7e-0.4 |
| | PAT vs AN2 | 0.38 | 3.8e-08 |
| modularity | PAT vs AN1 | 0.30 | 2.7e-0.3 |
| | PAT vs AN2 | 0.45 | 5.4e-10 |
| strong components | PAT vs AN1 | 0.56 | 1.5e-11 |
| | PAT vs AN2 | 0.49 | 1.1e-13 |

# References


1 Csardi, G. & Nepusz, T. The igraph software package for complex network research. *InterJournal* **Complex Systems**, 1695 (2006).
