# Supplementary material for: Microbial community organization designates distinct pulmonary exacerbation types and predicts treatment outcome in cystic fibrosis
Source: Nat Commun. 2024 Jun 7;15:4889. doi: 10.1038/s41467-024-49150-y (PMC11161516; doi:10.1038/s41467-024-49150-y)
Supplement: Supplementary file 3 — Reporting Summary [file 41467_2024_49150_MOESM3_ESM.pdf]

nature portfolio

Corresponding author(s): Stefanie Widder

Last updated by author(s): May 5, 2024

Reporting Summary

Nature Portfolio wishes to improve the reproducibility of the work that we publish. This form provides structure for consistency and transparency in reporting. For further information on Nature Portfolio policies, see our Editorial Policies and the Editorial Policy Checklist.

Statistics

For all statistical analyses, confirm that the following items are present in the figure legend, table legend, main text, or Methods section.

n/a	Confirmed	
	x	The exact sample size (*n*) for each experimental group/condition, given as a discrete number and unit of measurement
x		A statement on whether measurements were taken from distinct samples or whether the same sample was measured repeatedly
	x	The statistical test(s) used AND whether they are one- or two-sided<br>*Only common tests should be described solely by name; describe more complex techniques in the Methods section.*
	x	A description of all covariates tested
	x	A description of any assumptions or corrections, such as tests of normality and adjustment for multiple comparisons
	x	A full description of the statistical parameters including central tendency (e.g. means) or other basic estimates (e.g. regression coefficient) AND variation (e.g. standard deviation) or associated estimates of uncertainty (e.g. confidence intervals)
	x	For null hypothesis testing, the test statistic (e.g. *F*, *t*, *r*) with confidence intervals, effect sizes, degrees of freedom and *P* value noted<br>*Give P values as exact values whenever suitable.*
x		For Bayesian analysis, information on the choice of priors and Markov chain Monte Carlo settings
x		For hierarchical and complex designs, identification of the appropriate level for tests and full reporting of outcomes
	x	Estimates of effect sizes (e.g. Cohen's *d*, Pearson's *r*), indicating how they were calculated

Our web collection on statistics for biologists contains articles on many of the points above.

Software and code

Policy information about availability of computer code

Data collection	No software was used for data collection
Data analysis	R version 4.1.1 was used for data annotation and analysis. The R script collection and input data for this study is hosted at GitHub https://github.com/swidder/pex_types [10.5281/zenodo.11110106]. Large input data for code execution are hosted at Zenodo [https://zenodo.org/records/11109986].

For manuscripts utilizing custom algorithms or software that are central to the research but not yet described in published literature, software must be made available to editors and reviewers. We strongly encourage code deposition in a community repository (e.g. GitHub). See the Nature Portfolio guidelines for submitting code & software for further information.

Data

Policy information about availability of data

All manuscripts must include a data availability statement. This statement should provide the following information, where applicable:

- Accession codes, unique identifiers, or web links for publicly available datasets
- A description of any restrictions on data availability
- For clinical datasets or third party data, please ensure that the statement adheres to our policy

The generated sequencing data (FASTq files) are available as NCBI BioProjects under the accession numbers PRJNA987026[https://www.ncbi.nlm.nih.gov/bioproject/PRJNA987026], PRJNA520924 [https://www.ncbi.nlm.nih.gov/bioproject/PRJNA520924], and PRJNA611611 [https://www.ncbi.nlm.nih.gov/bioproject/PRJNA611611]. Source data for all figures are provided with this paper in Supplementary information/Source Data file. Moreover, data are presented disaggregated

by sex of the sample donor for every figure in the Source Data file. Furthermore, a list of ASVs as they appear in ASV groups and PEx types was added to Supplementary information/Source Data file. Sample data containing meta information for analyzed samples are similarly provided in Supplementary information/ Source Data/Sample Data.

Research involving human participants, their data, or biological material

Policy information about studies with human participants or human data. See also policy information about sex, gender (identity/presentation), and sexual orientation and race, ethnicity and racism.

Reporting on sex and gender	Self-reported
Reporting on race, ethnicity, or other socially relevant groupings	These data were not collected.
Population characteristics	11 adults with cystic fibrosis, five females, six males with mean age of 35.3 years and a range of 22-52 years. further characteristics by subjects, samples and time series are detailed in table 1.
Recruitment	Participants were recruited from among adults receiving care at the University of Michigan CF Care Center. Inclusion was conducted on expressed interest to participate in the study to avoid self-selection bias. To obtain participant's informed consents, individuals met face to face with a member of the study team before or after a regularly scheduled clinic appointment. The study team member reviewed the consent form with the individual in its entirety and answered any questions. Signatures of the individual and study team member were obtained on two copies of the consent form, one to go home with the individual and the other for study records. Information on participation compensation is not available.
Ethics oversight	This observational cohort study was approved by the University of Michigan Institutional Review Board (HUM00037056) on March 17, 2011 and renewed annually between 2011-2020.

Note that full information on the approval of the study protocol must also be provided in the manuscript.

Field-specific reporting

Please select the one below that is the best fit for your research. If you are not sure, read the appropriate sections before making your selection.

[x] Life sciences [ ] Behavioural & social sciences [ ] Ecological, evolutionary & environmental sciences

For a reference copy of the document with all sections, see nature.com/documents/nr-reporting-summary-flat.pdf

Life sciences study design

All studies must disclose on these points even when the disclosure is negative.

Sample size	11 adults with cystic fibrosis provided 880 sputum samples; of these were five participants were females and six males. All source data were disaggregated by sex of donors and are available as Source Data file complying with SAGE. No statistical method was used to predetermine sample size. Numbers of subjects and time series were selected based on availability of at least 60% of days during a time interval of 60 days cumulating in PEx.
Data exclusions	Subjects were selected from a larger dataset based on the availability of near-daily sputum samples (i.e., a sample available from at least 60% of days) that spanned 60 days-periods of clinical stability culminating with a PEx epsiode. Samples obtained during acute PEx treatment and 3 weeks thereafter (recovery) were excluded from the study. Inclusion criteria and exact numbers of samples and networks are detailed in two supporting tables (table S1, table S2).
Replication	Data derived from an observational prospective cohort study, no experiments were conducted or replicated.
Randomization	PEx groups were identified using non-standard sample descriptors, followed by ordination and K-mer clustering. The effect sizes of covariates were determined using PERMANOVA (partial omega2, Figure 1A). Microbiota were grouped into five groups, these are pathogens, anaerobes, facultative anaerobes, aerobes and unknown oxygen requirements.
Blinding	Blinding was not performed as data derived from an observational prospective cohort study.

Reporting for specific materials, systems and methods

We require information from authors about some types of materials, experimental systems and methods used in many studies. Here, indicate whether each material, system or method listed is relevant to your study. If you are not sure if a list item applies to your research, read the appropriate section before selecting a response.

Materials & experimental systems

n/a	Involved in the study
[x]	[ ] Antibodies
[x]	[ ] Eukaryotic cell lines
[x]	[ ] Palaeontology and archaeology
[x]	[ ] Animals and other organisms
[x]	[ ] Clinical data
[x]	[ ] Dual use research of concern
[x]	[ ] Plants

Methods

n/a	Involved in the study
[x]	[ ] ChIP-seq
[x]	[ ] Flow cytometry
[x]	[ ] MRI-based neuroimaging
